# The influence of frequently consumed beverages and snacks on dental erosion among preschool children in Saudi Arabia

**DOI:** 10.1186/s12937-017-0307-9

**Published:** 2017-12-11

**Authors:** Yousef H. Al-Dlaigan, Laila A. Al-Meedania, Sukumaran Anil

**Affiliations:** 1 Division of Pediatric Dentistry, Department of Pediatric Dentistry and Orthodontics, College of Dentistry, King Saud University, P.O. BOX 60169, Riyadh, 11545 Saudi Arabia; 2 Division of Pediatric Dentistry, Department of Pediatric Dentistry and Orthodontic Sciences, College of Dentistry, King Saud University, P.O. BOX 60169, Riyadh, 11545 Saudi Arabia; 3 Dental Biomaterials Research Chair, Dental Health Department, College of Applied Medical Sciences, King Saud University, Riyadh, Saudi Arabia

**Keywords:** Dental erosion, Children, Preschool, Dental, Diet, Beverages, Snacks

## Abstract

**Background:**

To determine the prevalence of dental erosion and its association to commonly used beverages and snacks among 3 to 5 year old preschool children in Riyadh, Saudi Arabia.

**Methods:**

Three hundred eighty-eight preschool children between 3 and 5 years old were selected from 10 different schools using a cluster random sample selection; there were 184 (47%) boys and 204 (53%) girls. The surfaces of each tooth were examined for erosion, and the level of tooth wear was recorded. Data on the frequently used beverages and snacks were obtained by questionnaires completed by the parents of the preschool children.

**Results:**

Among the 388 children examined, 47% exhibited low erosion, 10% exhibited moderate erosion and 4% exhibited severe erosion. There was no statistically significant difference between boys and girls in terms of the prevalence of erosion. Sixty percent of the children regularly consumed juice drinks. Among daily consumers, 84% of children showed erosion prevalence with strongly significant association (*p* < 0.005). Holding the drink in the mouth also showed a significant association with erosion (*p* < 0.02).

**Conclusion:**

It was concluded that an association was found between the prevalence of dental erosion and the frequency of citrus and carbonated juice consumed by the preschool children in Saudi Arabia.

## Background

Dental erosion (DE) is an important cause of tooth damage in children. DE is the loss of tooth structures such as enamel and dentine by a chemical process that does not involve bacteria. The extent of damage may range from a barely noticeable loss of surface luster that is evident on clean, dry enamel to the partial or the complete exposure of dentine with the appearance of a characteristic yellow color observable through the thin overlying enamel [1]. Dental erosion is caused by a chemical process (acids) without bacterial involvement, whereas attrition and abrasion are mechanical processes. The acids responsible for dental erosion can be of intrinsic or extrinsic origin [2]. Dental erosion may progress rapidly in the primary teeth compared to the permanent teeth due to the thinner enamel layer in primary teeth [3, 4]. Dental erosion may lead to dental hypersensitivity, altered occlusion, eating difficulties, poor esthetics, pulp exposure and dental abscesses [5].

The prevalence of dental erosion is higher in older children and is easier to detect [6]. The UK National Diet and Nutrition Survey reported that up to 10% of one and a half year old children showed signs of dental erosion on the labial surfaces of their primary teeth [7]. A study of 5-year-old children in the UK revealed a dental erosion prevalence of 53% [8]. According to the England, Wales, and Northern Ireland Children’s Dental Health Survey (2013), the prevalence of dental erosion reaching dentine on the surface of primary teeth was 17%; 58% of 5-year-old children presented with dental erosion reaching the enamel or dentine [9]. Additionally, studies have reported a wide range of prevalence of dental erosion (0.5% to 80%) in different countries in the world [5, 10–13].

Earlier studies have shown that boys exhibited more erosive lesions of the teeth than girls [14–17]. In Saudi Arabia, only a few studies have been carried out on the prevalence of dental erosion in primary teeth. Al-Majed et al. [18] studied both primary and permanent teeth in Riyadh and reported a dental erosion prevalence of 34% in 5 to 6 year old children and 26% in 12- to 14-year-old children. Another study conducted in Jeddah preschool children showed a similar dental erosion prevalence of 31% in primary incisors [19].

The consumption of acidic fruits, fruit juices, and carbonated drinks is directly correlated to dental erosion in children and adolescents [3, 5, 18]. Nayak et al. [20] found that frequent consumption of beverages significantly correlated with dental erosion, with a higher prevalence of dental erosion occurring in 5-year-old children who consumed the beverages daily. The most frequently consumed fruit juices and soft drinks commonly contain potentially damaging acids such as fruit acids and phosphoric acid [21].

The timing of the consumption of acidic beverages plays a critical role in the occurrence of dental erosion. Using a feeding bottle at night is highly associated with dental erosion [3, 18]. The reduction of saliva during bed time diminishes oral clearance. Therefore, an acidic diet will cause more destruction to tooth structure at night [22]. There is a general lack of awareness about dental erosion compared to other dental disorders. It is therefore important to assess the prevalence of dental erosion in preschool children, especially keeping in mind that erosion in primary teeth is a strong predictor of dental erosion in permanent teeth [23].

Prevalence studies on dental erosion in children must take into account the types of beverages, the frequency of beverage consumption, and the associations between the two. In Saudi Arabia, the consumption of soft drinks is high among children [24, 25]. There is a scarcity of literature on the prevalence of erosion and the etiologic factors leading to erosion. Therefore, the objective of this study was to determine the prevalence of dental erosion and investigate the dietary intake patterns of 3- to 5-year-old preschool children in Riyadh, Saudi Arabia, and to determine the relationship, if any, between dental erosion and dietary intake in these children.

## Methods

The questionnaire included general information on the child’s age and gender as well as dietary habits: food types (fruit, candy, sandwiches, donuts, ice cream, cake) and consumption of various drinks (citrus juice, mixed fruit juice, fresh fruit juice, flavored milk, plain milk). The clinical examination was carried out at the children’s schools (after receiving consent from the parents) using disposable mouth mirrors, explorers, gauze and tweezers, gloves, masks, protective eye glasses, gowns, a reclining chair and a portable light. The study was conducted in Riyadh Saudi Arabia, during August 2015 to February 2016.Each tooth surface was examined for dental erosion utilizing index criteria similar to criteria used in UK National Survey of Child Dental Health by O’Brien (1994) [26] (Table 1). The tooth surfaces (i.e., buccal/labial, palatal/lingual and incisal/occlusal), were examined for the loss of enamel surface characteristics and/or the exposure of dentin or pulp. Mesial and distal surfaces were excluded from the examination due to the close contact area especially between posterior teeth.

**Table 1:** Dental erosion index criteria [26]

Code 0	Normal
Code 1	Enamel only – loss of enamel surface gives characteristic of a smooth, glossy appearance with shallow chipped enamel.
Code 2	Enamel and dentin – loss of enamel, exposing dentin
Code 3	Enamel, dentin and pulp – extensive loss of enamel and dentin with pulpal exposure.
Code 9	Assessment cannot be made – when the tooth is either missing or has a large restoration depend on a score measure.

The children were classified into one of two groups based on tooth surface scores [14]. In cases of doubt, the lower score was assigned on the chart: Any child with a score of 0 was placed in the no erosion group; any child with a score of 1 on at least one tooth surface was placed in the low erosion group; any child with a score of 2 on at least one tooth surface was placed in the moderate erosion group; and any child with a score of 3 on at least one tooth surface was placed in the severe erosion group.

Another classification system for all of the children was based on their individual dental erosion status:

- Any child with no erosion on any tooth surface was placed in the “Group without Erosion,” and
- Any child with erosion on at least one surface or more than one surface was placed in the “Group with Erosion”.

The calibration studies were performed to assess intra- and inter-examiner reproducibility. The intra-examiner reproducibility assessed on a group of 10 children between 3 and 5 years of age yielded a weighted Kappa static value (0.95). The inter-examiner reproducibility study involved a different group of 20 children of a similar age; this showed a (0.80) weighted Kappa static value. These values indicated a high level of agreement in the assessment of dental erosion.

Of the 398 kindergarten schools with a total of 23,300 children, the required sample size to specify the probability of achieving statistical significance with margin of error at 5% level (confidence level of 95%) was calculated. The power analysis indicated that a sample of 378 children would be required for this study. This study utilized stratified random sampling to achieve an acceptable estimation of dental erosion prevalence in each stratum, with minimal sample random error. A total of 388 children were recruited for the clinical examinations. The participants included 184 (47%) boys and 204 (53%) girls aged three to five years, randomly selected from ten different kindergartens, two public and eight private schools because 80% of the preschool children in Riyadh attend private kindergartens. These ten kindergartens were randomly selected from various areas of the city of Riyadh (two kindergartens each from Central, Northern, Western, Southern and Eastern Regions). All data were analyzed using the Statistical Program for Social Sciences (SPSS - Version 20). Descriptive statistics methods were used to analyze the prevalence of dental erosion in addition to other variables related to dental erosion. The Chi-square test and Z-test were also utilized to analyze various independent factors related to age, gender and dietary intake.

## Results

Among the 388 children examined, 235 (61%) had erosion on at least one tooth surface. Low erosion confined only to enamel was observed in 182 (47%) children, and 37 (10%) children were diagnosed with moderate erosion. Pulp exposure due to severe erosion was seen in only 16 (4%) children (Table 2). Dental erosion was detected in 31.5% of all tooth surfaces. There was a comparable prevalence of dental erosion in boys and girls. Dental erosion significantly increased with the increasing age of children. Six percent of the 3-year-old children had dental erosion, followed by 30% and 64% of the 4 and 5-year-old children, respectively (*p* < 0.001). The most commonly affected teeth were maxillary teeth. The pronounced dental erosion (moderate and severe) appeared more often in the upper anterior teeth than in the posterior teeth. Data from the questionnaire revealed a higher level of mixed fruit drink consumption in 64% of the children with dental erosion compared to 36% of the children without dental erosion. The frequent consumption of citrus juice was associated with a high risk of erosion in 71% of the children. There was a statistically significant relationship between the consumption of citrus and fresh fruit juice and dental erosion (*p* < 0.04 *and p* < 0.02 respectively). The most consumed type of milk in this study was flavored milk; 220 children drank flavored milk, and 61% of those children exhibited tooth erosion, but this association was not statistically significant (*p* < 0.71) (Table 3). Erosion prevalence was significantly correlated to the daily consumption of juice drinks (*p* < 0.005). The daily consumption of juice drinks was reported in 84% of children with dental erosion. The proportion of children with erosion decreased to 77% and 65%, with a reduced frequency of consumption (1–6/week) and (1/week), respectively (Table 4). Regarding the type of snacks preferred by children, there was a significant difference in the intake of fruit between the groups with and without erosion (*p* < 0.01). Table 3 shows an association between dental erosion and type of beverages and snacks. Table 3 shows that the majority of erosion-free children did not consume a large volume of juice and showed a decrease in the frequency of consumption. Drinking without a straw was associated with erosion in 61% of children compared to children who used feeder cups (51% of the children had erosion) or a straw (53% of children had erosion). Direct swallowing of beverages was associated with the lowest prevalence of erosion (56% of children. Holding the drink for either a short or long time in the mouth increased the prevalence of erosion. This habit was significantly associated with erosion prevalence (*p* < 0.02). Table 4 presents the dental erosion and dietary habits.

**Table 2:** The prevalence of different levels of dental erosion in 388 preschool children

Level of erosion	Boys		Girls		Total	
	Number	%	Number	%	Number	%
No erosion (Code0)	69	38	84	41	153	39
Low erosion (Code1)	85	46	97	48	182	47
Moderate erosion (Code2)	21	11	16	8	37	10
Severe erosion (Code 3)	9	5	7	3	16	4
Total	184	100	204	100	388	010

**Table 3:** The type and rate of drink and snack consumption by children with and without erosion

	Total number of children	Children with erosion		Children without erosion		Chi-square test: *P* value
		Number	%	Number	%	
Type of consumed drink						
Citrus juice (lemon / orange)	72	51	71	21	29	*p* < 0.04
Carbonated bottled fruit juice	166	106	64	60	36	*p* < 0.25
Fresh fruit juice	161	87	54	74	46	*p* < 0.02
Flavored milk	220	135	61	85	39	*p* < 0.71
Plain milk	122	71	58	51	42	*p* < 0.51
Type of preferred snack						
Fruit (orange, banana, apple)	164	88	60	76	40	*p* < 0.01
Candy	113	73	65	40	35	*p* < 0.29
Sandwich	117	68	58	49	42	*p* < 0.51
Donuts	85	59	69	26	31	*p* < 0.06
Ice cream	141	85	60	56	40	*p* < 0.93
Cake	136	79	58	57	42	*p* < 0.46

**Table 4:** Drinking frequency and swallowing habits of children with and without erosion

Way of drinking	Total number of children	Children with Erosion		Children without Erosion		Chi-square test: *P* value
		Number	%	Number	%	
Feeder cup	64	33	51	31	49	*p* < 0.10
Cup with straw	32	17	53	15	47	*p* < 0.36
Cup without straw	150	92	61	58	39	*p* < 0.80
All	151	100	66	51	34	*p* < 0.06
Way of swallowing						
Directly swallow	251	140	56	111	44	*p* < 0.00
Hold then swallow	137	93	68	44	32	*p* < 0.02
Frequency of juice drink consumption						
No consumption	156	70	45	86	55	*p* < 0.00
One per week consumption	166	109	65	57	35	*p* < 0.07
More than one (two to six per week) consumption	35	27	77	8	23	*p* < 0.03
Daily consumption	31	26	84	5	16	*p* < 0.005
Frequency of snack consumption						
None, in between snack	41	17	41	24	59	*p* < 0.00
Consumption 1–3 times	331	207	62	124	38	*p* < 0.05
Consumption, more than 3 times	16	11	69	5	31	*p* < 0.46

## Discussion

There are limited data regarding the prevalence of dental erosion in preschool children in the Middle East. A wide range of dental erosion prevalence in primary dentition has been reported in different countries around the world [5, 10, 13, 27]. The high prevalence of dental erosion among three to five year old children reported in our study was similar to previous findings reported by Murakami et al. [28]. Both studies used the same scoring index of O’Brian, but our results showed a slightly higher erosion prevalence. This discrepancy might be due to the different environment and the higher consumption of erosive drinks by children in the present study group. Al-Majed et al. [18] conducted a similar study on primary dentition for five to six year old children in Riyadh and reported that pronounced dental erosion (with dentine and pulp involvement) was seen in 34% of their sample. Most of the children (47%) with dental erosion in the present study presented with mild cases of erosion that were confined only to the enamel; in contrast, moderate and severe erosion was seen in less than 14% of the children.

The dental erosion prevalence increases significantly with increasing age [19]. In the present study, the prevalence of dental erosion was 6% in three year olds, 30% in four year olds, and 64% in five-year-old children. This was consistent with another study reporting that dental erosion prevalence increased with increasing age [19]. Regarding dental erosion in relation to gender, no statistically significant difference in the prevalence of dental erosion amongst boys and girls was detected. Epidemiological data in previous studies showed that boys were more prone to dental erosion [14–17]. The maxillary teeth were more affected by dental erosion than the mandibular teeth in primary dentition which is in agreement with previous studies [18, 22]. The position and cleaning action of the tongue may have a protective role against acidic attack, which may lead to a decrease in the risk of dental erosion to the lower teeth [29].

Citrus juices were associated with a high erosion prevalence among the children in our study. This observation is in agreement with earlier reports [30, 31]. Many published studies have shown a direct association between the consumption of carbonated drinks and dental erosion in children and adolescents [32]. Because these carbonated drinks have a high acid content, the frequent consumption of carbonated drinks can be considered a risk factor for dental erosion [33]. An association between dental erosion and the daily consumption of juice drinks was observed in the present study. The prevalence of dental erosion decreased with the reduced frequency of consumption. The consumption of flavored and plain milk didn’t show any correlation with dental erosion in this study. Milk is non-cariogenic, as the sugar present is lactose which is the least cariogenic of all mono- and disaccharides [34]. Milk also contains factors such as calcium phosphate and casein that protect against demineralization of enamel [35].

An increased contact time between acidic beverages and teeth leads to a pronounced drop in pH. Some studies recommend drinking acidic beverages with a straw by putting it towards the back of the oral cavity to prevent dental erosion [36, 37]. In the present study, little reduction in erosion prevalence among children drinking with a feeder cup or with a straw was observed, which is supported by previous studies [36, 37]. The 2004 study by Johansson et al., [38] showed that dental erosion was associated with drinking methods. Holding the drink longer in the mouth leads to a more pronounced pH drop, resulting in dental erosion. There was also a significant association observed between dental erosion and the way in which children swallowed drinks.

The effect of snacks such as fruit, candy, sandwich, donuts, ice cream and cakes consumed by children on dental erosion was also evaluated. There was no significant impact of any type of snack on dental erosion, except for fruit. A cross sectional study on dental erosion and its association with diet in 12-year-old Libyan schoolchildren showed that the frequency of consumption of fruit-based drinks and length of time taken to consume acidic drinks were the primary statistically significant positive risk factors for dental erosion [33]. One of the limitation of the study is that the commonly consumed beverages and snacks by the children in Saudi Arabia were included in this study.

## Conclusions

Based on this study it can be concluded that the consumption of citrus juices, fruit juices and carbonated beverages showed an association to the prevalence of dental erosion in preschool children in Saudi Arabia. Measures such as health education programme targeting teachers, parents and school children may be undertaken to control the dental erosion among children.

## Abbreviations

DE: Dental erosion

## References

[1] Jaeggi T, Lussi A (2014). Prevalence, incidence and distribution of erosion. Monogr Oral Sci.

[2] Schuurs A, Schuurs A: Erosion. In Pathology of the Hard Dental Tissues. Hoboken: Wiley; 2012. p.156–172.

[3] Al-Malik MI, Holt RD, Bedi R, Speight PM (2001). Investigation of an index to measure tooth wear in primary teeth. J Dent.

[4] Johansson AK, Sorvari R, Birkhed D, Meurman JH (2001). Dental erosion in deciduous teeth--an in vivo and in vitro study. J Dent.

[5] Luo Y, Zeng XJ, MQ D, Bedi R (2005). The prevalence of dental erosion in preschool children in China. J Dent.

[6] Wiegand A, Muller J, Werner C, Attin T (2006). Prevalence of erosive tooth wear and associated risk factors in 2-7-year-old German kindergarten children. Oral Dis.

[7] Friebe D. National Diet and nutrition survey: children aged 1 1/2 to 4 1/2 years. Volume 1: report of the diet and nutrition survey. Edited by J. R. Gregory, D. L. Collins, P. S. W. Davis, J. M. Hughes and P. C. Clarke. 391 pages, numerous figures and tables. HMSO, London 1995. Food Nahrung. 1996;40:48–8.

[8] Chadwick BL, White DA, Morris AJ, Evans D, Pitts NB (2006). Non-carious tooth conditions in children in the UK, 2003. Br Dent J.

[9] Todd JE, Dodd T. Children's Dental Health in the United Kingdom, 1983: A Survey Carried Out by the Social Survey Division of OPCS, on Behalf of the United Kingdom Health Departments, in Collaboration with the Dental Schools of the Universities of Birmingham and Newcastl. Stationery Office. 1985;

[10] Ayers KM, Drummond BK, Thomson WM, Kieser JA (2002). Risk indicators for tooth wear in New Zealand school children. Int Dent J.

[11] Kazoullis S, Seow WK, Holcombe T, Newman B, Ford D (2007). Common dental conditions associated with dental erosion in schoolchildren in Australia. Pediatr Dent.

[12] Mantonanaki M, Koletsi-Kounari H, Mamai-Homata E, Papaioannou W (2013). Dental erosion prevalence and associated risk indicators among preschool children in Athens, Greece. Clin Oral Investig.

[13] Moimaz SA, Araujo PC, Chiba FY, Garbin CA, Saliba NA (2013). Prevalence of deciduous tooth erosion in childhood. Int J Dent Hyg.

[14] Al-Dlaigan YH, Shaw L, Smith A (2001). Dental erosion in a group of British 14-year-old, school children. Part I: prevalence and influence of differing socioeconomic backgrounds. Br Dent J.

[15] van Rijkom HM, Truin GJ, Frencken JE, Konig KG, Van 't Hof MA, Bronkhorst EM, Roeters FJ (2002). prevalence, distribution and background variables of smooth-bordered tooth wear in teenagers in the hague, the Netherlands. Caries Res.

[16] Dugmore CR, Rock WP (2003). The progression of tooth erosion in a cohort of adolescents of mixed ethnicity. Int J Paediatr Dent.

[17] Bardsley PF, Taylor S, Milosevic A (2004). Epidemiological studies of tooth wear and dental erosion in 14-year-old children in north West England. Part 1: the relationship with water fluoridation and social deprivation. Br Dent J.

[18] Al-Majed I, Maguire A, Murray JJ (2002). Risk factors for dental erosion in 5-6 year old and 12-14 year old boys in Saudi Arabia. Community Dent Oral Epidemiol.

[19] Al-Malik MI, Holt RD, Bedi R (2002). Erosion, caries and rampant caries in preschool children in Jeddah, Saudi Arabia. Community Dent Oral Epidemiol.

[20] Nayak SS, Ashokkumar BR, Ankola AV, Hebbal MI (2010). Distribution and severity of erosion among 5-year-old children in a city in India. J Dent Child (Chic).

[21] Al-Dlaigan YH, Shaw L, Smith A (2001). Dental erosion in a group of British 14-year-old school children. Part II: influence of dietary intake. Br Dent J.

[22] Lussi A, Jaeggi T, Zero D (2004). The role of diet in the aetiology of dental erosion. Caries Res.

[23] Ganss C, Lussi A, Grunau O, Klimek J, Schlueter N (2011). Conventional and anti-erosion fluoride toothpastes: effect on enamel erosion and erosion-abrasion. Caries Res.

[24] Musaiger A, Zagzoog N (2014). Knowledge, attitudes and practices toward energy drinks among adolescents in Saudi Arabia. Glob J Health Sci.

[25] Wyne AH, Chohan AN, Al-Begomi R (2002). feeding and dietary practices of nursing caries children in Riyadh, Saudi Arabia. Odontostomatol Trop.

[26] O'Brien M (1994). Report of dental survey. Office of Population Censuses and Surveys. Children's Dental Health in the United Kingdom 1993.

[27] Linnett V, Seow WK (2001). Dental erosion in children: a literature review. Pediatr Dent.

[28] Murakami C, Oliveira LB, Sheiham A, Nahas Pires Correa MS, Haddad AE, Bonecker M (2011). risk indicators for erosive tooth wear in Brazilian preschool children. Caries Res.

[29] Manaf ZA, Lee MT, Ali NH, Samynathan S, Jie YP, Ismail NH, Bibiana hui Ying Y, Wei Seng Y, Yahya NA (2012). relationship between food habits and tooth erosion occurrence in Malaysian university students. Malays J Med Sci.

[30] de Melo MA, Passos VF, Lima JP, Parente GC, Rodrigues LK, Santiago SL (2015). Erosive potential of processed and fresh orange juice on human enamel. J Dent Child (Chic).

[31] Grando LJ, Tames DR, Cardoso AC, Gabilan NH (1996). In vitro study of enamel erosion caused by soft drinks and lemon juice in deciduous teeth analysed by stereomicroscopy and scanning electron microscopy. Caries Res.

[32] Al-Malik MI, Holt RD, Bedi R (2001). The relationship between erosion, caries and rampant caries and dietary habits in preschool children in Saudi Arabia. Int J Paediatr Dent.

[33] Sovik JB, Skudutyte-Rysstad R, Tveit AB, Sandvik L, Mulic A (2015). Sour sweets and acidic beverage consumption are risk indicators for dental erosion. Caries Res.

[34] Giacaman RA, Munoz-Sandoval C (2014). Cariogenicity of different commercially available bovine milk types in a biofilm caries model. Pediatr Dent.

[35] Reynolds EC, Johnson IH (1981). Effect of milk on caries incidence and bacterial composition of dental plaque in the rat. Arch Oral Biol.

[36] Imfeld T (1996). Dental erosion. Definition, classification and links. Eur J Oral Sci.

[37] Edwards M, Ashwood RA, Littlewood SJ, Brocklebank LM, Fung DE (1998). A videofluoroscopic comparison of straw and cup drinking: the potential influence on dental erosion. Br Dent J.

[38] Johansson AK, Lingstrom P, Imfeld T, Birkhed D (2004). Influence of drinking method on tooth-surface pH in relation to dental erosion. Eur J Oral Sci.

